# Effect of high-flow nasal cannula oxygen on diaphragmatic excursion and lung volumes determined by electrical impedance tomography

**DOI:** 10.1186/2197-425X-3-S1-A165

**Published:** 2015-10-01

**Authors:** S Perbet, S Bertran, B Longere, B Pereira, E Futier, JM Constantin

**Affiliations:** CHU Clermont-Ferrand, CHU Estaing, Clermont-Ferrand, France; CHU Clermont-Ferrand, Réanimation Adultes, Clermont-Ferrand, France; CHU Clermont-Ferrand, Biostatistics Unit, Clermont-Ferrand, France; CHU Clermont-Ferrand, Pôle Médecine Péri-Opératoire, Clermont-Ferrand, France

## Introduction

High Flow Nasal Cannulae (HNFC) is commonly used to improve oxygenation in critically ill patients. But it effects on diaphragm motion and end expiratory lung volumes (EELV) are not well described.

## Objectives

To assess and compare effects of different flows of HFNC and conventional facemask on the diaphragm excursion. Secondary outcomes were to assess effects on patient discomfort, PaO2/FiO2 ratio, EELI and TDI variation measured by electrical impedance tomography (EIT).

## Methods

Cross-over single prospective study on patients undergoing a major abdominal surgery and high respiratory risk assessed by ARISCAT score > 26 admitted in a surgical intensive care unit.

## Results

Twenty consecutive patients were included. The mean ARISCAT score was 41. The mean right diaphragmatic excursion was significantly increased with increasing inspiratory flow (baseline: 0.73 mm, 20l / min: 0.99 mm, 40l / min: 1.26 mm, 60l / min 1.51 mm, baseline. 0.66 mm, p < 0.0001). Linear regression analysis showed a dependent speed increase without ceiling effect. Lung volumes were also significantly increased compared to baseline, with increasing total flow (20l / min: 8%, 40 l / min: 16%, 60 l / min: + 24% baseline: p < 0.05) and equally PaO2 (baseline: 79 20l / min: 124, 40l / min: 152, 60l / min: 155, baseline. 81 mmHg, p < 0.0001). A significant decrease in respiratory rate was observed (baseline: 20.4, 20l / min 19.2, 40l / min: 17.2, 60 l / min: 16.7, baseline. 20.3 / min, p < 0.0001). No difference were noted in the modified score Borg and the right or left side.

## Conclusions

Application of progressive flow through HFNC improves diaphragmatic excursion, lung volumes and oxygenation while decreasing respiratory rate, speed-dependent manner. Its clinical impact remains to be seen in the population of high respiratory risk patients undergoing major abdominal surgery.

